# Dioxins reformation and destruction in secondary copper smelting fly ash under ball milling

**DOI:** 10.1038/srep22925

**Published:** 2016-03-15

**Authors:** Giovanni Cagnetta, Mohammed Mansour Hassan, Jun Huang, Gang Yu, Roland Weber

**Affiliations:** 1 State Key Joint Laboratory of Environment Simulation and Pollution Control (SKJLESPC), Beijing Key Laboratory of Emerging Organic Contaminants Control (BKLEOCC), School of Environment, POPs Research Center, Tsinghua University, Beijing 100084, P. R. China; 2 POPs Environmental Consulting, Lindenfirststrasse 23, 73527 Schwaebisch Gmuend, Germany

## Abstract

Secondary copper recovery is attracting increasing interest because of the growth of copper containing waste including e-waste. The pyrometallurgical treatment in smelters is widely utilized, but it is known to produce waste fluxes containing a number of toxic pollutants due to the large amount of copper involved, which catalyses the formation of polychlorinated dibenzo-*p*-dioxins and dibenzofurans (“dioxins”). Dioxins are generated in secondary copper smelters on fly ash as their major source, resulting in highly contaminated residues. In order to assess the toxicity of this waste, an analysis of dioxin-like compounds was carried out. High levels were detected (79,090 ng TEQ kg^−1^) in the ash, above the Basel Convention low POPs content (15,000 ng TEQ kg^−1^) highlighting the hazardousness of this waste. Experimental tests of high energy ball milling with calcium oxide and silica were executed to assess its effectiveness to detoxify such fly ash. Mechanochemical treatment obtained 76% dioxins reduction in 4 h, but longer milling time induced a partial *de novo* formation of dioxins catalysed by copper. Nevertheless, after 12 h treatment the dioxin content was substantially decreased (85% reduction) and the copper, thanks to the phenomena of incorporation and amorphization that occur during milling, was almost inactivated.

Polychlorinated dibenzo-*p*-dioxins and polychlorinated dibenzofurans (PCDD/Fs, commonly named “dioxins”) are two groups of unintentionally produced persistent pollutants (POPs) with 75 and 135 congeners, respectively. Some of these congeners are among the most toxic chemicals known. They interfere with the modulation of gene expression, which induces subsequent biochemical, cellular, and tissue modifications that result in several toxic effects^1^. Also other compounds with a similar chemical structure can have dioxin-like toxicity, such as 12 congeners of polychlorinated biphenyls (PCBs), which are designated as *dioxin-like* PCBs (*dl*-PCBs)^2^ and are included in the general term of “dioxin-like compounds”. The dioxin concentration is normally expressed as toxic equivalency (TEQ) values, where each congener with dioxin-like toxicity has been assigned a toxic equivalency factor (TEF) defined by World Health Organization expert group^3^.

Dioxins and dioxin-like compounds are chemically stable and persistent in the environment. They are recalcitrant to biodegradation and other natural attenuation processes while photochemical transformation of non-adsorbed molecules can effectively degrade these POPs^4^. They are mainly formed during combustions and other thermal processes^5^,^6^ and the largest historic release has been formed as by-products in the production of chlorinated organic compounds^7^,^8^,^9^,^10^,^11^. Natural sources of dioxins have been identified, e.g. volcanoes and forest fires^12^,^13^, but are of minor relevance compared to anthropogenic sources, as seen by the historic records in sediment cores^14^,^15^.

Dioxins were and are released from these sources into environmental compartments and accumulate in soil and sediments^10^,^16^ or were disposed in landfills and dump sites^11^,^17^,^18^.

The formation of PCDD/Fs in combustion processes has been comprehensively studied. Formation reactions take place in gas phase via precursors (chlorophenols or chlorobenzenes) or on ash particles by degradation of soot or polycyclic aromatic hydrocarbons (PAHs) (*de novo* synthesis) in the temperature range of 200–600 °C 6,19,20,21.

Metal oxides and chlorides are potent catalysts for dioxins formation^22^,^23^. Actually, the catalytic pathway is believed to be more relevant than the homogeneous reaction for dioxins generation and transition metals are known for their promoting effect; specifically, copper remarkably enhances the PCDD/Fs formation yield, either by *precursors* route or *de novo* synthesis^6^,^21^,^22^,^24^. The *de novo* pathway is extremely relevant in flue gas cooling section of incinerators, smelting processes in the secondary metal industry and range of other thermal processes^11^ having temperature zones between 200 and 600 °C and chloride, carbon and catalytic metals present. Here the fly ash (FA) amount and activity (depending on catalytic metals, chlorine and carbon content) play a key role in determining PCDD/Fs formation^25^.

In order to reduce or eliminate the production of POPs (including dioxins) and to prevent unintentional POPs formation and release by human activities 179 countries ratified the Stockholm Convention^26^.

The Convention encourages the utilization of non-combustion technologies capable to irreversibly destroy POPs. However, it has been highlighted that also some of these technologies can form PCDD/Fs^27^.

High energy ball milling (HEBM) is listed as a non-combustion technology for POPs destruction^28^. Mechanical energy is transferred by milling bodies (often stainless steel balls) into the milled materials resulting in chemical reactions. Such mechanochemical (MC) transformations can be achieved in special milling devices (e.g. planetary ball mills, stirred ball mills, etc.) at moderate bulk-temperatures (few tens K higher than room temperature), without solvents, and utilizing minimal processing without (or, at most, with very few) pre- and post-treatment operations. Hence HEBM is considered an environmentally friendly technology^29^.

HEBM is a promising technology for the destruction of chlorinated, brominated and fluorinated POPs to halides and amorphous/graphitic carbon^30^,^31^,^32^,^33^,^34^,^35^,^36^,^37^. An increase of the TEQ level in the milled material was once reported, due to dechlorination of PCDD/Fs with high chlorination degree into lowly chlorinated congeners with higher toxicity and TEF values^32^. Co-milling with cheap reagents, such as CaO, can however destroy and mineralize dioxins^38^. HEBM was also successfully employed to reduce PCDD/Fs content in FA collected from municipal^32^ and medical waste incinerators^39^,^40^.

HEBM treatment of waste with a relevant quantity of PCDD/Fs reformation catalyst, such as copper compounds, has never been investigated. However, adequate technologies for waste with high copper concentration and high dioxin levels above the Basel Convention low POPs content are needed for safe disposal or recovering of this enlarging material flow when considering the continuously developing global secondary copper production^41^.

Here the depletion of copper primary reserves, whereas world demand is increasing, has led to a growing importance of “secondary sources”, viz. metallurgical, industrial, consumer, and electronic waste. In particular, the rapid expansion of the electronic markets together with the short lifespan of electronic devices has determined a remarkable increase of e-waste production, demanding for adequate recovery processes. In many countries (e.g. China, EU, Japan) secondary sources treatment is mandatory to recover copper and other precious metals. Pyrometallurgical processing of secondary copper is widely utilized to this end, but it is characterized by a number of issues not prevalent in smelting of primary sources, e.g. specific gas handling/cleaning operations are required for capturing NO_x_, halogens, dioxins, etc^41^. Besides, secondary copper smelting FA is an hazardous waste with a tremendous potential for dioxin release and reformation due to the significant content of dioxins and dioxin *de novo* formation potential^42^,^43^. For this reason, direct thermal treatment of FA or its recycle in the smelter is not recommendable, because of direct release and fast reformation during the cooling phase of the flue gases, unless expensive emission control technologies are implemented.

In order to verify if FA containing large amounts of copper can be safely treated by HEBM to reduce dioxins content, laboratory tests were carried out in the present study demonstrating for the first time that dioxins can be formed in FA under HEBM conditions. In addition, the study demonstrates that with adequate duration and control, dioxins could be finally destroyed and reduced the formation potential, and the technology can be further tested and possibly used in real processes.

## Results and Discussion

### Fly ash characterization

Analysis of the untreated FA sample revealed extreme high TEQ levels of total dioxins of 79,090 ng TEQ kg^−1^, therefore higher than the low Basel POPs content of 15,000 ng TEQ kg^−1^. The individual amounts of PCDDs, PCDFs, and *dl*-PCBs were 13,160 ng TEQ kg^−1^, 61,090 ng TEQ kg^−1^, and 4,840 ng TEQ kg^−1^, respectively, with a predominance of high chlorinated congeners (Table 1).

The quantity of chlorobenzenes (PeCBz + HxCBz) was quite low, i.e. 313,000 ng kg^−1^ (Table 1).

Reagents for the *de novo* synthesis (i.e. carbon and chlorides), as well as metal catalysts (i.e. copper, iron, etc.), were abundant in the FA (Table 2). The high level of chlorides (2.6%) and copper (5.7%) resulted in the formation of highly chlorinated congeners^23^,^42^,^43^. Copper not only acts as catalyst for condensation, dechlorination, etc., but also as a shuttle for chlorine between the gas phase and the solid carbonaceous material^25^,^44^. Besides, the prevalence of PCDFs respect to PCDDs is common in the *de novo* pathway. Furans are mostly generated from pre-existing ring structures in the carbonaceous matter, which are simultaneously formed, and rarely by single-ring condensation, that on the contrary is relevant in the PCDDs formation^44^.

These extreme high levels of dioxin toxicity above the provisional Basel low POPs content highlight that secondary copper smelting FA deserve particular attention in their management and recycling. Currently the common practice is the recycling of these ashes in the smelting furnace to recover the precious metals, which might lead to further formation and release of dioxins.

### Optimization of the co-milling mixture

The destruction of PCDD/Fs by MC was demonstrated previously with pure CaO under relatively harsh conditions, namely with a large amount of co-milling reagent (i.e. 200:1 CaO-to-dioxin ratio) and providing high mechanical energy intensity to the reaction mixture (i.e. 700 rpm planetary disk rotation speed)^38^. Milder conditions were not enough to achieve a complete conversion in few hours treatment^32^,^40^. Nevertheless, it was ascertained that MC destruction of chlorinated pollutants with CaO is roughly independent from milling intensity and, when low intensities are provided, longer milling times are sufficient to obtain complete destruction of pollutants^45^.

In order to reduce the quantity of reagents, in the present work, we tested a mixture of CaO and SiO_2_, which has been found effectual to destroy a number of chlorinated molecules^37^,^46^,^47^,^48^,^49^. Both oxides are present in FA to some extent and contribute to the MC destruction of dioxins^40^. The FA from the copper smelter has only low concentration of these elements, with a CaO concentration of 1.0% and SiO_2_ of 0.5% (Table 2).

**Figure 1:**
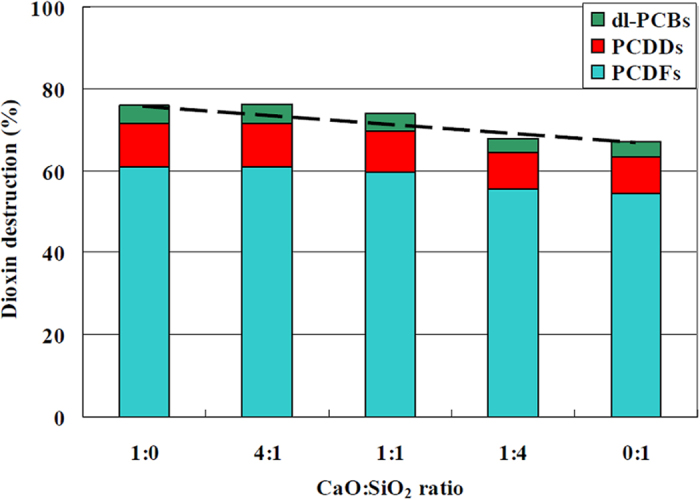
Dioxins destruction percentages after 4 h ball milling with different co-milling reagent mixtures.

The effectiveness of CaO to destroy dioxins is confirmed; after 4 h milling 76% of pollutants are removed from FA sample co-milled with addition of CaO:SiO_2_ = 1:0 (Fig. 1). Compared to CaO, the addition of silica (i.e. CaO:SiO_2_ = 0:1) had a slightly lower destruction capability but also achieving a total dioxins destruction efficiency of 67%.

The congener distribution in the residue after treatment (Table 3) did not show a change of PCDDs, PCDFs, and *dl*-PCBs fingerprints, i.e. their relative composition was almost constant.

Chlorobenzenes were not assessed in the co-milling reagent optimization because of their low initial concentration (Table 1).

The good performance of CaO can be explained by its double role, namely the provision of electrons for dioxins dechlorination and carbonization, as well as the subtraction of chlorine from the reaction mixture by CaCl_2_ formation^38^,^50^. Ikoma *et al.*^51^ proposed that during milling the oxide centres of the CaO lattice are activated by the mechanical energy, so oxygen atoms donate an electron to carbon atoms. This phenomenon is supposed to happen independently from neighbour atoms of the carbon. If a C−Cl bond exists, the electron obtained from the oxide is transferred to the chlorine, which detaches from the molecule as chloride. In this degradation pathway a C−O bond is formed with an unpaired electron on the oxygen (i.e. a radical). The reaction progresses through radical mechanism to mineralization of pollutants into amorphous carbon and chlorides.

The degradation mechanism of silica is less clear. It is known that SiO_2_ during milling is a plasma-former: Electron-rich fresh surfaces are generated because of the homolytic Si−O bond breaking in the silica crystals^52^. The subsequent interaction of activated silica and pollutants is not ascertained. Probably, Si· and Si−O· radicals attack and bind chlorine atoms of pollutant molecules, detaching them and starting the radical degradation of the carbon skeleton until its final destruction to amorphous carbon^37^.

The CaO:SiO_2_ = 4:1 mixture destruction percentage was the same as with pure CaO, but it diminishes as the silica content increases (Fig. 1). Such behaviour can be explained considering the relative hardness of the two reagents. During milling, the softer material (i.e. CaO, Mohs hardness 3.5) is rapidly comminuted compared to the harder component (i.e. SiO_2_, Mohs hardness 7, and the other FA constituents), and covers the latter^53^. Hence, CaO, when utilized in relatively high amount (i.e. CaO:SiO_2_ = 4:1 and 1:1), is not hindered by a “dilution” with silica because it covers completely the other particles and is almost the only reagent in contact with the pollutant molecules.

### MC destruction of PCDD/Fs, PCBs, PeCBz, and HxCBz

Although the efficiencies of pure CaO and CaO:SiO_2_ = 4:1 are the same (i.e. 76.0 and 76.2, respectively), the latter was chosen for studying the time dependency of MC destruction because the two components are normally present in FA.

**Figure 2:**
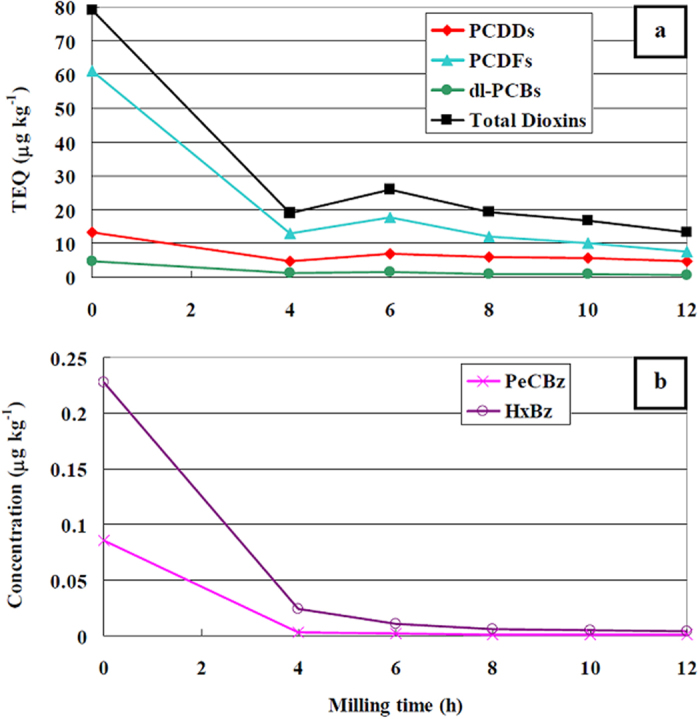
MC destruction of dioxins (**a**) and chlorobenzenes (**b**) with CaO:SiO_2_ = 4:1 as co-milling reagent.

The results were unexpected. After 4 h milling, which resulted in 76% pollutants destruction, the dioxins concentration increased in the experiment of 6 h compared to 4 h and then further decreased at 8 h and longer milling time (Fig. 2a). Congener analysis showed no significant change in dioxins fingerprint (Table 4).

The observed reformation of dioxins (mainly PCDD/Fs) can be explained by the presence of copper, which catalyse the reformation reaction. According to the MC hot-spot theory, during milling, when a ball hits another ball or the jar wall compressing a small amount of powder, on the contact surface the temperature rises up to thousands of K in a microscopic area (~1 μm^2^) for a very short time (~10^−9^ s)^54^. Near the contact area the temperature increase is less and last longer. Under these conditions and in the presence of high amount of copper (5.8%), chloride (2.6%) and carbon (30.8%), *de novo* reformation reaction seems to occur.

Very recently, Wang *et al.*^43^ verified on FA from secondary copper smelting facility, with high content of this metal, an extreme high reformation rate when it was heated at 350 °C under continuous air flow: Here PCDD/Fs concentration increases more than one hundred times in 10 min. PCDD/Fs reformation during HEBM is slower than under heating, being a solid state reaction occurring at environmental bulk-temperature. The milling procedure does not create ideal conditions for dioxins formation in the FA, but the local increment of the temperature on FA particle surfaces due to milling and the good oxygenation of the milled material determined by the continuous mixing seem sufficient that *de novo* synthesis occurs.

Despite the occurrence of the reformation reaction for long milling time (after 6 h), the destruction reaction prevailed and a decreasing trend of the dioxins content is observed (Fig. 2a). This time trend indicates that conditions for the reformation reaction changed during HEBM. The concentration of PeCBz and HxCBz, i.e. two possible dioxins precursors, shows a rapid and substantial decrease (Fig. 2b). Concerning the reagents involved in the *de novo synthesis* (i.e. carbon and chlorine), they are present in relatively high quantity and their modification seems less likely compared to the sensitive catalyst copper and the copper-chloride system.

Copper compounds are likely trapped into the inorganic matrix and/or transformed into an amorphous material, so their catalytic effect seems to decrease at longer duration. Both effects on metals were observed during HEBM. Heavy metals were immobilized in 3 different kinds of soils by mechanical treatment^55^. Immobilization was caused by entrapment of heavy metals into aggregates, their solid diffusion into the crystalline reticulum of soil particles, as well as by the formation of fresh surfaces (through particle breakage) onto which heavy metals may be irreversibly adsorbed. Another possible effect is the amorphization of the FA inorganic components, which is a well-known effect of intensive ball milling^53^. The destruction of copper compounds structure may cause the loss of catalytic activity of the metal. It is probable that a regular lattice is required to adsorb carbonaceous species and promote their oxidation and rearrangement in dioxin form. However, to the best of our knowledge, such problem has never been investigated. If such affirmation is correct (i.e. copper compounds need a crystal structure to explicate their catalytic effect), then catalyst amorphization may be responsible of the activity loss. These two phenomena (i.e. entrapment and amorphization) can inactivate the copper in the FA, which reasonably will no longer be active for dioxin formation. This is a key result of the FA mechanical treatment that is capable not only to destroy dioxins in the waste, but also to transform such waste in a less active material for possibly further recycling or for a safer disposal. This needs further assessment in respect to *de novo* formation if recycled in smelters and by leaching tests for possible disposal.

In the present work, 12 h HEBM was not enough to obtain a complete destruction of dioxins. Longer milling time is sufficient to conclude the MC reaction^45^, but future investigation, in order to reduce energy cost, should focus on specific co-milling reagents that are effective at stoichiometric amount and react with a faster kinetic (which were already tested with other halogenated pollutants^34^).

### Kinetic analysis

The decrease and increase of dioxins TEQ over the experimental time is very peculiar and needed further analysis. Actually it indicates that at the start of the experiment the degradation of the PCDD/Fs present is rather fast and the reformation of PCDD/Fs is slower in the beginning. Furthermore the newly formed PCDD/Fs seem to be adsorbed in areas with lower destruction rate. The newly formed dioxins seem less accessible compared to the largest part of the original dioxins. Since these congeners are not chemically different (the congener pattern did not change significantly, as shown in Table 4), they might be better adsorbed to the carbon. Also for the original PCDD/Fs present, the accessibility to destruction is most likely different, depending on the adsorption location. A similar phenomenon is observed for extraction of PCDD/Fs from FA, which need, for instance, acid treatment to break-up the matrix, otherwise a fraction of PCDD/Fs cannot be extracted.

This dependence on the adsorption location explains also that the observed destruction rates are independent from chlorination degree. Here the destruction rate is different for PCDDs, PCDFs, and *dl*-PCBs because of the diversity of their interactions with the FA carbonaceous component; such interactions are not determined significantly by the chlorination degree.

Concerning catalyst, as discussed in the previous paragraph, copper promotes the formation and destruction reaction of dioxins. Also here the availability changes over time and, with increased mixing/milling with CaO and SiO_2_, copper compounds get entrapped into the inorganic matrix due to aggregation/agglomeration of FA particles. Consequently, they are inactivated over time and cannot explicate their catalytic function.

These considerations suggest that under milling the following reactions take place:


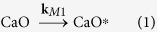



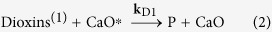



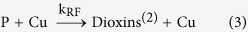



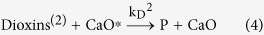



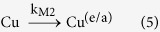


Equation (1) describes the activation of CaO to the state CaO^*^ (k_M1_ is the kinetic constant for the mechanical activation of the oxide); equation (2) summarizes the destruction reaction of Dioxins^(1)^ originally present on the FA (with kinetic constant k_D1_), which regenerates CaO and gives origin to a group of products P that mainly includes chlorides and carbon; such products P can reform new Dioxins^(2)^ (equation (3), kinetic constant k_RF_) by means of the catalytic effect of copper oxides, chlorides, etc. (briefly indicated as Cu); Dioxins^(2)^, which behave differently from Dioxins^(1)^ as explained above, are destroyed by activated CaO^*^ (equation (4), with a kinetic constant k_D2_ supposed to be different from k_D1_); the entrapment/amorphization of Cu consumes the catalyst of reaction equation (3) (equation (5), kinetic constant k_M2_).

The following differential equation system derives from chemical equations (1, 2, 3, 4, 5):


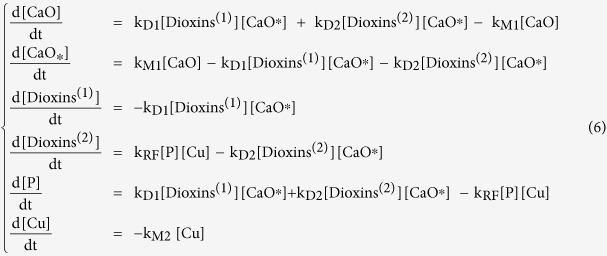


Boundary conditions were chosen taking into account the initial composition of the milled reaction mixture. Specifically, CaO concentration was simply calculated considering that 1.6 g of it were added to the milled mixture (4 g final weight). Copper compounds (Cu) amount was inferred from FA composition (Table 2). The initial concentration value of the compounds that are responsible of dioxins *de novo* synthesis (P) was considered null, even if chlorides and carbon were already present; tentative non-zero initial values were evaluated, but, unless the figure was very low, fitting of experimental data was not good.


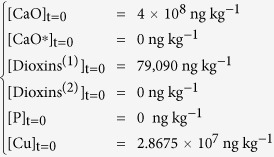


In order to reduce in some way the degrees of freedom of the system (6), milling constants k_M1_ and k_M2_ were assumed to be equal, considering that milling effects should be similar for all components in the jar.

After best-fitting, kinetic constants with the following values were obtained:


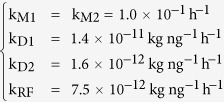


**Figure 3:**
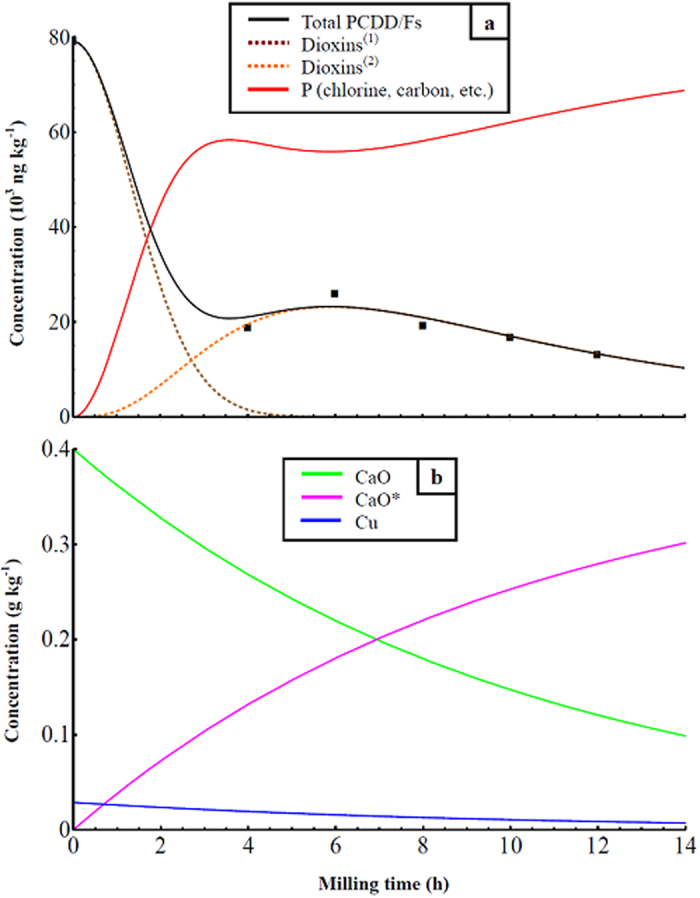
Numerical solution for differential equations system (6), showing trends of: total dioxins with experimental points, their components (i.e. Dioxins^(1)^ and Dioxins^(2)^), and their degradation products P (**a**); reagents (i.e. CaO and its activated form CaO^*^), and catalyst (i.e. copper compounds Cu) (**b**).

The numerical solution of the system (6) is presented in Fig. 3. Kinetic analysis highlights some interesting outcomes of the present work.

The trend of total dioxins depicted in Fig. 3a fits satisfactorily to the experimental data. Dioxins reformation rate constant k_RF_ is approximately half of the destruction rate constant of original dioxins k_D1_, but the destruction rate constant of the newly formed pollutants k_D2_ is 5 times lower than the reformation constant. Hence, reformation is faster than degradation of the newly formed dioxins, being more difficult to destroy due to their adsorption location. Results support the idea that, in average, dioxins originally present in the FA sample and newly formed dioxins, although with similar chemical fingerprint, interact in a different manner with the carbon. However, this interpretation of the experimental results needs further investigations.

Dioxins are reformed due to the copper, but the latter is “inactivated” over time by ball milling. Its declining trend (Fig. 3b) together with the progressive destruction reaction (equation (4)) are responsible for the final overall destruction of the dioxins.

The reformation of PCDD/Fs during ball milling demonstrates that emerging non-combustion technologies need to be assessed for the formation of unintentional POPs (including dioxins) when POPs and other chlorinated pollutants are destroyed. Only the ascertainment of the irreversible destruction of POPs and unintentional by-products assures an overall detoxification of the treated waste^27^. This needs to be assessed also when there are changes in the waste composition. For example, in previous studies dioxin formation under HEBM was not observed for different matrices, including FA. However these FA samples did not contain such high copper content and did not have such extreme *de novo* formation potential as ashes from secondary copper production.

In addition to dioxin precursors, also the presence of copper compounds, as well as other catalytic metals, in waste materials must be considered cautiously, and a thorough investigation should be conducted to assess unintentional formation of dioxins during the ball milling or other treatment.

Also a kinetic analysis should be performed to determine appropriate destruction time. In our case prolonged milling is needed to treat secondary copper smelting FA, because this can overcompensate the reformation and allows overall dioxins destruction and detoxification. After assessing the essential reaction time, an assessment of the engineering implications needs to be performed to decide on practicability of the reaction time and necessary modifications for possible commercial application.

### Proposed degradation and reformation mechanism

**Figure 4:**
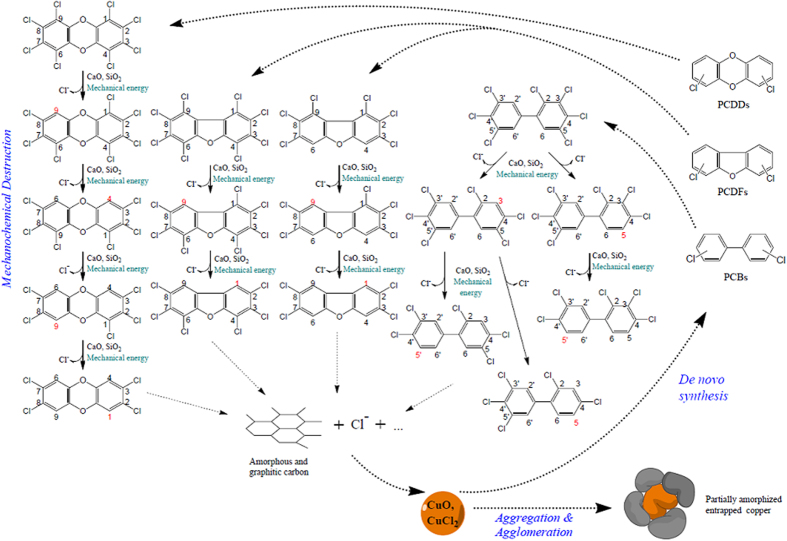
Hypothesized MC destruction pathways of PCDD/Fs and *dl*-PCBs, their reformation, and catalyst deactivation.

Mechanisms that likely take place during HEBM of secondary copper smelting FA with CaO and SiO_2_ are briefly summarized in Fig. 4.

The two co-milling reagents are activated by mechanical energy provided by the mill. CaO is forced to act as a Lewis base in the dechlorination of dioxins, as well as in the carbonization reaction; SiO_2_ is fractured, exposing electron-rich fresh surfaces that transform pollutants carbon skeleton into carbon and dechlorinate it as a collateral effect. In the present work, CaO almost certainly plays the major role in dioxins destruction, as similarly observed in thermal degradation of PCDD/F on FA^56^,^57^.

Amorphous and graphitic carbon and chlorides are the final products of MC destruction. The second beneficial effect of HEBM is the subtraction from the reaction mixture of copper compounds. They are probably entrapped into the solid matrix of FA, or are partially amorphized, so such compounds cannot further catalyse the dioxins reformation reaction.

Regarding dioxins transformation, additional details can be inferred by chemical speciation and how congeners’ reduction percentages (respect to their initial concentration) slightly vary during milling (Table 4). Data suggest that a minor dechlorination takes place in parallel to the destruction of the carbon skeleton, as also observed in thermal destruction of PCDD/Fs on FA^56^,^57^. Low chlorinated molecules display a certain increasing trend, while high chlorinated compounds have a higher decrease. Such finding is coherent with the results obtained by Peng *et al.*^39^ on medical waste incinerator FA ball milled with CaO, who observed similar destruction percentages for all PCDD/Fs homologues, with a moderate preference for the highly chlorinated congeners. Actually, also in the present work perchlorinated homologues exhibit slightly higher destruction percentages (i.e. >90% for both OCDD and OCDF) compared to the lower chlorinated congeners.

However, Peng *et al.*^39^ did not detect in their milling experiments any preference between peri-positions (viz. 1, 4, 6, 9) and lateral-positions (viz. 2, 3, 7, 8) in PCDD/Fs; also in thermal dehalogenation on FA no difference was observed^56^. On contrary, we notice that products deriving from dechlorination of peri-positions show a relative increase (Table 4). Specifically (as shown in Fig. 4), the OCDF, after losing two chlorine atoms in positions 1 and 9 (which seem to be the most favourable for Cl detachment), is dechlorinated to the 2,3,4,6,7,8-HxCDF, which shows a degradation percentage of only 35% in 4 h milling. In addition, a considerable higher reduction of 1,2,3,7,8,9-HxCDF (87% in 4 h) and 1,2,3,7,8-PeCDF (84% in 4 h) (as well as 84% of 2,3,4,7,8-PeCDF, not shown in Fig. 4) corroborates the hypothesis that positions 1 and 9 are weak and easy to dechlorinate under MC conditions. This was also observed for the thermal PCDF pattern and the thermal destruction of PCDF due to steric hindrance of the crowding, when 1- and 9-positions are substituted by chlorine^56^.

Also OCDD is preferably dechlorinated in peri-position (Fig. 4). Here it is partly dechlorinated to 1,2,3,7,8,9-HxCDD (OCDD and 1,2,3,4,6,7,8-HpCDD exhibit reductions of 94% and 79%, respectively, after 4 h milling), while 1,2,3,7,8,9-HxCDD shows a degradation percentage lower than the average (that is 63% in 4 h). The latter compound can be dechlorinated to 1,2,3,7,8-PeCDD, which is quite slowly degraded (with 55% reduction).

PCBs dechlorination pathway cannot be inferred clearly from the few *dioxin-like* congeners determined, and a detailed congener-specific analysis is needed for. Nonetheless, the *dl*-PCB data suggest that the *meta-* position is possibly more favourable for dechlorination (Fig. 4). In particular 2,3,3′,4,4′,5,5′-HpCB shows a quite high reduction percentage, i.e. higher than the average of all other measured PCBs for milling times longer than 4 h (Table 4). This finding is in accordance with the observed preference for the *meta*- and *para*-position in the thermal degradation of PCBs on FA^56^.

The observed slight preference for peri-positions in PCDD/Fs dechlorination under HEBM conditions is also corroborated by quantum-mechanical estimations^32^. This needs to be considered and assessed for the employment of MC technology at full-scale. However our and other ball milling experiments at laboratory-scale achieved satisfying degradation for 2,3,7,8-TCDD and 2,3,7,8-TCDF. As emphasized by Weber^27^, monitoring of dioxins levels in full-scale destruction is required to ensure that the lower chlorinated and higher toxic congeners are not formed in destruction technologies involving (partly) dechlorination. This requires further attention and investigations to assess the feasibility of HEBM as an alternative technology for dioxins destruction. Overall the study has shown the potential of HEBM to destroy dioxins also in this increasingly important waste and recycling materials. Therefore the technology has the potential to facilitate further recovery of copper from this waste.

## Materials and Methods

### Milling experiments

Calcium oxide (CaO, reagent grade, Beijing Chemical Works, China) was pre-treated at 800 °C for 2 h. Silica (SiO_2_, Beijing Modern Eastern Fine Chemicals, China) was used in the experiment without pre-treatment. Both materials were employed as components of the co-milling reagent mixture.

The FA was collected from an industrial secondary copper smelting furnace in China. The sample was dry and homogeneous and was used in the experiment as-obtained.

X-ray fluorescence spectrometry (XRF) (XGT-7200, Horiba, Japan) was employed to determine the inorganic composition of the FA.

Milling tests were carried out in a planetary ball mill (XMQ-0.4L, Kexi, China), at 275 rpm (jar-to-planetary disk rotation speed ratio was equal to −2); 180 g stainless steel balls (Ø 5 mm) were loaded with 2 g co-milling reagent mixture and 2 g FA in each stainless steel jar (Ø 40 mm, volume 70 cm^3^). Reagent mixtures were prepared with different CaO:SiO_2_ weight ratios, as follows: 1:0, 4:1, 1:1, 1:4, and 0:1.

### Materials for organic pollutants quantification

All the solvents used for extraction and clean-up procedures were of pesticide residue analysis grade, and were purchased from Duksan (South Korea). Florisil solid-phase exchange cartridges (1000 mg, 6 mL), multi-layer silica-gel columns, and silica-gel dispersed carbon columns (Kanto Chemicals Co., Japan) were utilized for clean-up procedures. ^13^C-labeled standard solutions of PCDDs, PCDFs, PeCBz, HxCBz (Cambridge Isotope Laboratories, USA) and *dl*-PCBs (Wellington Laboratories, Canada) were used as internal standards. Unlabeled standard solutions and 1,2,4,5-tetrabromobenzene were purchased from AccuStandard (USA).

### Extraction, clean-up, and analysis of PCDD/Fs and dl-PCBs

After the milling tests, the FA sample (0.1 g) was spiked with ^13^C-labeled PCDD/Fs and *dl*-PCBs as internal quantification standards.

Digestion with hydrochloric acid was conducted to destroy the inorganic cladding structure. HCl was added to the samples until the foam formation stopped. Then the sample was washed with deionized water, followed by ulterior washing with acetone to remove residual HCl and water. The aqueous/acetone phase was liquid-liquid extracted with dichloromethane. The FA sample was dried at room temperature and extracted by Soxhlet extractor with 300 mL toluene for 16 h. The Soxhlet extract and the liquid-liquid extract were combined and the combined solvents were evaporated in a rotary evaporator and dissolved into hexane. The sample was finally treated with concentrated sulphuric acid by shaking in a separating funnel to remove interferences.

The hexane supernatant was concentrated and cleaned up with a multi-layer silica-gel column, eluted with 200 mL hexane. The sample was further fractionated using a silica-gel dispersed carbon column (1 g), eluted with 25 mL hexane, followed by 40 mL dichloromethane:hexane 1:3. After, the carbon column was switched up and down, and eluted with 50 mL toluene. Finally, the whole concentrated elute was spiked with ^13^C-labeled internal standards as syringe spike and concentrated to 50 μL by a pressurized gas blowing concentrator (Shuaien, China).

PCDD/Fs and *dl*-PCBs were quantified by HRGC-HRMS (6890N network gas chromatograph Agilent, USA coupled with a JEOL JMS-800D high-resolution mass spectrometer). The quantification was made by the isotope dilution method and the relative response factor of calibration standards. A BPX-DXN column (60 m × 0.25 mm × 0.25 μm, SGE Analytical Science, Australia) was used for PCDD/Fs determination, with the following temperature program: 130 °C initial oven temperature, hold for 1 min, ramped at 150 °C/min to 210 °C, at 3 °C/min up to 310 °C, and at 50 °C/min up to 320 °C with a final hold time of 10 min. For *dl*-PCBs determination, a RH-12ms column (60 m × 0.25 mm × 0.25 μm, InventX, USA) was utilized, with the following temperature program: 130 °C for 1 min, 15 °C/min to 210 °C for 0 min 30 °C/min to 310 °C for 0 min, 50 °C/min to 320 °C for 10 min. Highly purified helium was used as carrier gas with constant pressure of 25.4 psi. The injector, the interface, and the ion source temperatures were set at 300 °C. The HRMS was operated both in electron impact (38 eV) and selected ion monitoring mode at a resolution >10000 (10% valley).

Dioxins concentrations are expressed as TEQ, according to WHO (2005) TEF^3^.

### Extraction, clean-up, and analysis of penta- and hexa-chlorobenzene

A portion of milled FA (0.1 g) was spiked with PeCBz and HxCBz 250 μL of 1 μg mL^−1 13^C-labeled internal standards solution. After, the sample underwent solvent extraction by Soxhlet extractor (Eyela, Japan) with 300 mL of toluene for 16 h. The extracted solution was concentrated to 0.5 mL by rotary evaporator, diluted again in 30 mL hexane, and concentrated once more to remove almost all toluene. Concentrated extracts were cleaned up in Florisil cartridge (activated by 5 mL hexane:acetone 1:1 and, subsequently, by 10 mL hexane). The elution was concentrated to 0.5 mL, and added with 50 μL of 1 ng mL^−1^ tetrabromobenzene solution as syringe spike.

PeCBz and HxCBz quantification was carried out by GC-MS (QP 2010-plus, Shimadzu, Japan) with 1 mL/min highly purified helium as carrier gas, and 30 m DB-5MS column (Agilent, USA); the temperature program was: 80 °C for 1 min, 100 °C/min to 200 °C, hold for 1 min, 30 °C/min to 300 °C, hold for 1 min; temperatures of the injector, the interface, and the ion source were set at 280 °C, 270 °C, 250 °C, respectively. The MS was operated both in electron impact and selected ion monitoring mode.

### Quality assurance and quality control

Quality assurance and quality control were conducted with the method blank, duplicate samples and recoveries of labelled compounds. The deviation of duplicate samples was within 30%. The recoveries of ^13^C-labeled internal quantification standards were in compliance with Chinese HJ 77.3-2008 and EPA 1613 methods^58^,^59^.

The detection limits of the instruments, methods, and samples were checked and confirmed regularly. A method blank was conducted during each batch of samples to confirm the blank value had no statistical significance. The detection limits of dioxin congeners for 0.1 g FA sample were in the range 15–100 pg g^−1^.

## Additional Information

**How to cite this article**: Cagnetta, G. *et al.* Dioxins reformation and destruction in secondary copper smelting fly ash under ball milling. *Sci. Rep.* **6**, 22925; doi: 10.1038/srep22925 (2016).

## Figures and Tables

**Table 1:** PCDDs, PCDFs, and *dl*-PCBs concentration in the untreated FA sample.

Compound	Detected concentration (ng kg^−1^)	TEF	Equivalent quantity (ng TEQ kg^−1^)
2,3,7,8-TCDD	960	1	960
1,2,3,7,8-PeCDD	5,500	1	5,500
1,2,3,4,7,8-HxCDD	11,000	0.1	1,100
1,2,3,6,7,8-HxCDD	23,000	0.1	2,300
1,2,3,7,8,9-HxCDD	14,000	0.1	1,400
1,2,3,4,6,7,8-HpCDD	170,000	0.01	1,700
OCDD	200,000	0.001	200
2,3,7,8-TCDF	9,500	0.1	950
1,2,3,7,8-PeCDF	26,000	0.05	1,300
2,3,4,7,8-PeCDF	53,000	0.5	27,000
1,2,3,4,7,8-HxCDF	84,000	0.1	8,400
1,2,3,6,7,8-HxCDF	74,000	0.1	7,400
2,3,4,6,7,8-HxCDF	89,000	0.1	8,900
1,2,3,7,8,9-HxCDF	34,000	0.1	3,400
1,2,3,4,6,7,8-HpCDF	28,000	0.01	2,800
1,2,3,4,7,8,9-HpCDF	54,000	0.01	540
OCDF	400,000	0.001	400
3,3′,4,4′-TeCB (#77)	13,000	0.0001	1.3
3,4,4′,5-TeCB (#81)	11,000	0.0003	3.3
3,3'4,4′,5-PeCB (#126)	39,000	0.1	3,900
3,3′,4,4′,5,5′-HxCB (#169)	31,000	0.03	930
2,3,3′,4,4′-PeCB (#105)	18,000	0.00003	0.54
2,3,4,4′,5-PeCB (#114)	10,000	0.00003	0.3
2,3′,4,4′,5-PeCB (#118)	9,300	0.00003	0.28
2′,3,4,4′,5-PeCB (#123)	3,300	0.00003	0.1
2,3,3′,4,4′,5-HxCB (#156)	40,000	0.00003	1.2
2,3,3′,4,4′,5′-HxCB (#157)	26,000	0.00003	0.78
2,3′,4,4′,5,5′-HxCB (#167)	11,000	0.00003	0.33
2,3,3′,4,4′,5,5′-HpCB (#189)	63,300	0.00003	1.9
**Total Dioxins**			**79,090**
PeCBz	86,000	–	–
HxCBz	227,000	–	–

**Table 2:** Inorganic composition of the FA from XRF analysis (elemental and oxide weight percentage).

Element	Percentage (%)	Oxide	Percentage (%)
C	30.7966	CO_2_	58.4290
Zn	26.8868	ZnO	14.1702
P	14.4681	P_2_O_5_	18.7752
O	13.6555		
Cu	5.7109	CuO	3.1266
Sn	2.8203	SnO_2_	1.8630
Cl	2.5953	Cl	1.3842
Ca	1.2816	CaO	0.9271
Br	0.4761	Br	0.1714
Si	0.388	SiO_2_	0.4758
Pb	0.2227	PbO	0.0863
Fe	0.1526	Fe_2_O_3_	0.1065
K	0.1395	K_2_O	0.0877
S	0.1099	SO_3_	0.1470
Mn	0.1019	MnO	0.0645
Al	0.0854	Al_2_O_3_	0.0937
Mg	0.0649	MgO	0.0631
Cr	0.0229	Cr_2_O_3_	0.0165
Ni	0.0126	NiO	0.0077
Mo	0.0084	MoO_3_	0.0045

**Table 3:** Concentrations of dioxins after 4 h HEBM with various co-milling reagent mixtures.

Compound (ng kg^-1^)	CaO:SiO_2_ reagent ratio									
	1:0		4:1		1:1		1:4		0:1	
	TEQ	Reduction %	TEQ	Reduction %	TEQ	Reduction %	TEQ	Reduction %	TEQ	Reduction %
2,3,7,8-TCDD	280	70.83	290	69.79	290	69.79	350	63.54	350	63.54
1,2,3,7,8-PeCDD	2,500	54.55	2,500	54.55	2,700	50.91	3,100	43.64	3,200	41.82
1,2,3,4,7,8-HxCDD	350	68.18	360	67.27	370	66.36	460	58.18	460	58.18
1,2,3,6,7,8-HxCDD	740	67.83	730	68.26	850	63.04	1,000	56.52	1,000	56.52
1,2,3,7,8,9-HxCDD	500	64.29	520	62.86	560	60.00	700	50.00	680	51.43
1,2,3,4,6,7,8-HpCDD	360	78.82	360	78.82	400	76.47	510	70.00	510	70.00
OCDD	13	93.50	13	93.50	16	92.00	21	89.50	20	90.00
2,3,7,8-TCDF	240	74.74	220	76.84	240	74.74	310	67.37	290	69.47
1,2,3,7,8-PeCDF	210	83.85	210	83.85	240	81.54	290	77.69	330	74.62
2,3,4,7,8-PeCDF	4,200	84.44	4,200	84.44	4,500	83.33	5,400	80.00	5,700	78.89
1,2,3,4,7,8-HxCDF	2,200	73.81	2,200	73.81	2,500	70.24	3,100	63.10	3,400	59.52
1,2,3,6,7,8-HxCDF	1,800	75.68	1,800	75.68	2,000	72.97	2,400	67.57	2,600	64.86
2,3,4,6,7,8-HxCDF	1,200	86.52	1,200	86.52	1,000	88.76	1,300	85.39	1,300	85.39
1,2,3,7,8,9-HxCDF	2,200	35.29	2,200	35.29	2,600	23.53	3,200	5.88	3,200	5.88
1,2,3,4,6,7,8-HpCDF	680	75.71	680	75.71	750	73.21	940	66.43	970	65.36
1,2,3,4,7,8,9-HpCDF	140	74.07	140	74.07	150	72.22	200	62.96	190	64.81
OCDF	20	95.00	20	95.00	23	94.25	30	92.50	30	92.50
3,3′,4,4′-TeCB	0.45	65.38	0.4	69.23	0.41	68.46	0.49	62.31	0.52	60.00
3,4,4′,5-TeCB	1.2	63.64	0.96	70.91	1.2	63.64	1.3	60.61	1.4	57.58
3,3'4,4′,5-PeCB	1,100	71.79	1,000	74.36	1,100	71.79	1,600	58.97	1,400	64.10
3,3′,4,4′,5,5′-HxCB	250	73.12	210	77.42	270	70.97	360	61.29	330	64.52
2,3,3′,4,4′-PeCB	0.17	68.52	0.17	68.52	0.17	68.52	0.23	57.41	0.21	61.11
2,3,4,4′,5-PeCB	0.11	63.33	0.09	70.00	0.11	63.33	0.13	56.67	0.14	53.33
2,3′,4,4′,5-PeCB	0.093	66.79	0.087	68.93	0.1	64.29	0.12	57.14	0.12	57.14
2′,3,4,4′,5-PeCB	0.033	67.00	0.033	67.00	0.036	64.00	0.045	55.00	0.048	52.00
2,3,3′,4,4′,5-HxCB	0.36	70.00	0.28	76.67	0.39	67.50	0.51	57.50	0.48	60.00
2,3,3′,4,4′,5′-HxCB	0.24	69.23	0.21	73.08	0.28	64.10	0.33	57.69	0.33	57.69
2,3′,4,4′,5,5′-HxCB	0.12	63.64	0.11	66.67	0.13	60.61	0.15	54.55	0.15	54.55
2,3,3′,4,4′,5,5′-HpCB	0.51	73.16	0.45	76.32	0.57	70.00	0.69	63.68	0.66	65.26
**Total Dioxins**	**18,986**	**75.99**	**18,856**	**76.16**	**20,562**	**74.00**	**25,275**	**68.04**	**25,964**	**67.17**

**Table 4:** Dioxins and chlorobenzenes concentrations during HEBM with CaO:SiO_2_ = 4:1.

Compound (ng kg^−1^)	Milling time									
	4 h		6 h		8 h		10 h		12 h	
	TEQ	Reduction %	TEQ	Reduction %	TEQ	Reduction %	TEQ	Reduction %	TEQ	Reduction %
2,3,7,8-TCDD	290	69.79	390	59.38	540	43.75	610	36.46	700	27.08
1,2,3,7,8-PeCDD	2,500	54.55	3,700	32.73	3,500	36.36	3,500	36.36	2,800	49.09
1,2,3,4,7,8-HxCDD	360	67.27	490	55.45	400	63.64	330	70.00	210	80.91
1,2,3,6,7,8-HxCDD	730	68.26	950	58.70	710	69.13	600	73.91	470	79.57
1,2,3,7,8,9-HxCDD	520	62.86	720	48.57	620	55.71	470	66.43	380	72.86
1,2,3,4,6,7,8-HpCDD	360	78.82	550	67.65	320	81.18	250	85.29	200	88.24
OCDD	13	93.50	15	92.50	8.1	95.95	6	97.00	4.5	97.75
2,3,7,8-TCDF	220	76.84	300	68.42	320	66.32	310	67.37	320	66.32
1,2,3,7,8-PeCDF	210	83.85	300	76.92	260	80.00	220	83.08	170	86.92
2,3,4,7,8-PeCDF	4,200	84.44	5,700	78.89	4,500	83.33	3,900	85.56	3,300	87.78
1,2,3,4,7,8-HxCDF	2,200	73.81	2,900	65.48	2,000	76.19	1,600	80.95	1,100	86.90
1,2,3,6,7,8-HxCDF	1,800	75.68	3,500	52.70	1,700	77.03	1,400	81.08	1,000	86.49
2,3,4,6,7,8-HxCDF	1,200	86.52	1,000	88.76	660	92.58	530	94.04	360	95.96
1,2,3,7,8,9-HxCDF	2,200	35.29	3,000	11.76	2,100	38.24	1,700	50.00	1,100	67.65
1,2,3,4,6,7,8-HpCDF	680	75.71	900	67.86	520	81.43	420	85.00	300	89.29
1,2,3,4,7,8,9-HpCDF	140	74.07	160	70.37	96	82.22	78	85.56	52	90.37
OCDF	20	95.00	22	94.50	10	97.50	7.8	98.05	6	98.50
3,3′,4,4′-TeCB	0.4	69.23	0.38	70.77	0.41	68.46	0.39	70.00	0.36	72.31
3,4,4′,5-TeCB	0.96	70.91	0.9	72.73	0.93	71.82	0.9	72.73	0.63	80.91
3,3'4,4′,5-PeCB	1,000	74.36	1,200	69.23	880	77.44	780	80.00	640	83.59
3,3′,4,4′,5,5′-HxCB	210	77.42	240	74.19	150	83.87	140	84.95	96	89.68
2,3,3′,4,4′-PeCB	0.17	68.52	0.18	66.67	0.15	72.22	0.14	74.07	0.11	79.63
2,3,4,4′,5-PeCB	0.09	70.00	0.09	70.00	0.072	76.00	0.069	77.00	0.048	84.00
2,3′,4,4′,5-PeCB	0.087	68.93	0.11	60.71	0.11	60.71	0.099	64.64	0.093	66.79
2′,3,4,4′,5-PeCB	0.033	67.00	0.045	55.00	0.039	61.00	0.036	64.00	0.029	71.00
2,3,3′,4,4′,5-HxCB	0.28	76.67	0.42	65.00	0.29	75.83	0.25	79.17	0.17	85.83
2,3,3′,4,4′,5′-HxCB	0.21	73.08	0.22	71.79	0.15	80.77	0.12	84.62	0.099	87.31
2,3′,4,4′,5,5′-HxCB	0.11	66.67	0.13	60.61	0.099	70.00	0.093	71.82	0.075	77.27
2,3,3′,4,4′,5,5′-HpCB	0.45	76.32	0.54	71.58	0.3	84.21	0.26	86.32	0.18	90.53
**Total Dioxins**	**18,856**	**76.16**	**26,040**	**70.18**	**19,297**	**78.67**	**16,854**	**80.94**	**13,210**	**84.76**
PeCBz	3.03	96.47	1.89	97.80	1.26	98.53	0.893	98.96	0.618	99.28
HxCBz	23.63	89.61	10.92	95.20	5.80	97.45	5.16	97.73	3.480	98.47
